# Accuracy of saliva for SARS-CoV-2 detection in outpatients and their household contacts during the circulation of the Omicron variant of concern

**DOI:** 10.1186/s12879-023-08271-3

**Published:** 2023-05-05

**Authors:** Guilherme Calvet, Maria Ogrzewalska, Wagner Tassinari, Lusiele Guaraldo, Paola Resende, Trevon Fuller, Stephanie Penetra, Michele Borges, Anielle Pina-Costa, Ezequias Martins, Isabella Moraes, Heloisa Santos, Luana Damasceno, Fernando Medeiros-Filho, Otavio Espindola, Fernando Mota, Valéria Nacife, Alex Pauvolid-Corrêa, Jimmy Whitworth, Chris Smith, Marilda Siqueira, Patrícia Brasil

**Affiliations:** 1 Acute Febrile Illnesses Laboratory, Evandro Chagas National Institute of Infectious Diseases, Oswaldo Cruz Foundation, Av. Brasil, 4365, Manguinhos, Rio de Janeiro, Rio de Janeiro 21045-900 Brazil; 2 Oswaldo Cruz Institute, Oswaldo Cruz Foundation, Rio de Janeiro, Rio de Janeiro Brazil; 3 SARS-CoV-2 National Reference Laboratory for the Brazilian Ministry of Health (MoH) and Regional Reference Laboratory in Americas for the Pan-American Health Organization (PAHO/WHO), Rio de Janeiro, Brazil; 4 Federal Rural University of Rio de Janeiro, Rio de Janeiro, Rio de Janeiro Brazil; 5 Departments of Clinical Research and Epidemiology and Public Health, London School of Hygiene and Tropical Medicine, London, UK

**Keywords:** COVID-19, SARS-CoV-2, Omicron, Saliva, Accuracy

## Abstract

**Background:**

While nasopharyngeal (NP) swabs are considered the gold standard for severe acute respiratory coronavirus 2 (SARS-CoV-2) real-time reverse transcriptase-polymerase chain reaction (RT-PCR) detection, several studies have shown that saliva is an alternative specimen for COVID-19 diagnosis and screening.

**Methods:**

To analyze the utility of saliva for the diagnosis of COVID-19 during the circulation of the Omicron variant, participants were enrolled in an ongoing cohort designed to assess the natural history of SARS-CoV-2 infection in adults and children. Sensitivity, specificity, positive predictive value (PPV), negative predictive value (NPV), and Cohen’s kappa coefficient were calculated to assess diagnostic performance.

**Results:**

Overall, 818 samples were collected from 365 outpatients from January 3 to February 2, 2022. The median age was 32.8 years (range: 3–94 years). RT-PCR for SARS-CoV-2 was confirmed in 97/121 symptomatic patients (80.2%) and 62/244 (25.4%) asymptomatic patients. Substantial agreement between saliva and combined nasopharyngeal/oropharyngeal samples was observed with a Cohen’s kappa value of 0.74 [95% confidence interval (CI): 0.67–0.81]. Sensitivity was 77% (95% CI: 70.9–82.2), specificity 95% (95% CI: 91.9–97), PPV 89.8% (95% CI: 83.1–94.4), NPV 87.9% (95% CI: 83.6–91.5), and accuracy 88.5% (95% CI: 85.0-91.4). Sensitivity was higher among samples collected from symptomatic children aged three years and older and adolescents [84% (95% CI: 70.5–92)] with a Cohen’s kappa value of 0.63 (95% CI: 0.35–0.91).

**Conclusions:**

Saliva is a reliable fluid for detecting SARS-CoV-2, especially in symptomatic children and adolescents during the circulation of the Omicron variant.

## Background

On November 26, 2021, the World Health Organization (WHO) designated lineage B.1.1.529 a variant of concern (VOC) named Omicron, on the advice of WHO’s Technical Advisory Group on Virus Evolution [1]. At the beginning of January 2022, Rio de Janeiro, Brazil, experienced a considerable increase in cases of COVID-19. Genomic sequencing studies carried out by the COVID-19 Oswaldo Cruz Foundation (Fiocruz) Genomic Surveillance Network and other institutions suggested that the Omicron variant was responsible for 96% of these cases (http://www.genomahcov.fiocruz.br/dashboard-en/) [2].

While nasopharyngeal (NP) swabs are considered the gold standard for SARS-CoV-2 real-time reverse transcriptase-polymerase chain reaction (RT-PCR) detection, several studies have shown that saliva is an alternative specimen for COVID-19 diagnosis and screening [3–6], including for asymptomatic persons and outpatients [7, 8]. Collecting saliva is non-invasive and is better tolerated and accepted than swabs [7]. As a result, there is a reduced infection risk for healthcare workers and decreased personal protective equipment usage because direct interaction between healthcare workers and patients can be avoided [9, 10]. Yee et al. observed that the performance of saliva and NP swabs were comparable for symptomatic and asymptomatic pediatric patients [9]. In addition, a study showed a 100% positive agreement for the Omicron variant in saliva swabs compared to paired mid-turbinate swabs [11].

We report the accuracy of unstimulated whole saliva (UWS) through drooling compared to combined nasopharyngeal and deep oropharyngeal (OP) swabs in outpatients with suspected COVID-19 and their household contacts. We also compared the distribution of Cycle threshold (Ct) values in the UWS and combined NP/OP of asymptomatic and symptomatic patients.

## Methods

Patient information and clinical samples were derived from an open, prospective cohort study designed to assess the natural history of SARS-CoV-2 infection in adults and children. Contacts residing in the same domicile as the index case were offered enrolment in the study. Asymptomatic and symptomatic adults, adolescents, and children were recruited at the Evandro Chagas National Institute of Infectious Diseases (INI) and Germano Sinval Faria Health Centre [12], located in the metropolitan region of Rio de Janeiro during the study period. Residents of the community of Manguinhos, a region with less favorable socioeconomic characteristics, were recruited at the Germano Sinval Faria Health Centre. On the other hand, the participants recruited at the outpatient clinic (INI) came from throughout the metropolitan region of Rio de Janeiro, which includes a number of municipalities that are far from the capital and have a different sociodemographic profile.

Regular home visits according to a pre-defined schedule were made to collect samples (serum, saliva, and naso-oral swabs specimens) and complete the study forms. We collected clinical data, including sociodemographic information, date of symptom onset (if any of the following were present: cough, shortness of breath, fever, chills, headache, loss of taste or smell, fatigue, muscle aches, sore throat, xerostomia, nasal congestion or rhinorrhea, arthralgia, prostration, abdominal pain, nausea, vomiting or diarrhea, and skin rashes), and type of sample collected (UWS vs. NP/OP). The present manuscript described the results of a selection of the cohort participants with an available paired specimen collected during their regular study visits or unscheduled study visits in case a participant developed COVID-19 symptoms during the circulation of Omicron in January 2022. Some participants had more than one paired sample collection during the period.

NP/OP and UWS were collected on the same visit. Nurses collected NP/OP swabs. UWS was collected, under nurses’ supervision, by asking the participant to accumulate saliva (at least 1–2 mL) in the mouth for one minute and then drooling into a sterile container without coughing or clearing their throats. UWS was collected only for children who drool saliva (aged three years and older). Combined NP/OP swabs were placed in a Falcon tube with 3 mL of Viral Transport Medium (VTM). Samples were transferred on the same day in a refrigerated bag to the Laboratory of Respiratory Viruses and Measles, a national reference laboratory for SARS-CoV-2 RT-PCR testing.

On receipt at the national reference laboratory, a suspension was prepared consisting of UWS and VTM that had a final volume of 2 mL. Before preparing aliquots for extractions, all samples were vortex-homogenized for 30 s. The viral RNA was extracted automatedly using 300 µl of the sample and Perkin-Elmer Chemagic machine/chemistry. SARS-CoV-2 positive cases were confirmed by real-time RT–PCR assays using the SARS-CoV-2 Molecular E/RP Kit (Biomanguinhos, Rio de Janeiro, Brazil) [13] based on the protocol previously designed by Corman et al. [14]. Amplifications were conducted in the ABI7500 platform using the following conditions: reverse transcription (50 °C, 15 min), reverse transcriptase inactivation and DNA polymerase activation (95 °C, 2 min), followed by 45 cycles of DNA denaturation (95 °C, 20 s) and annealing–extension (58 °C, 30 s). All samples with sigmoid curves crossing the threshold line up to cycle 40 were considered positive. Negative and positive controls were included in each extraction and real-time RT–PCR batch.

Cycle threshold (Ct) values of RT-PCR targeting the E gene were recorded for all positive results. Frequencies and percentages were reported for categorical variables. Sensitivity, specificity, positive predictive value (PPV), and negative predictive value (NPV), with a 95% confidence interval (CI), were calculated to assess diagnostic performance [15]. The Cohen’s kappa coefficient was used to estimate the agreement between the UWS and combined NP/OP swabs results as a reference gold standard. We used Wilcoxon’s signed-rank test to compare the Ct values of UWS and combined NP/OP samples.

Furthermore, we compared Ct values between asymptomatic and symptomatic RT-PCR confirmed patients and between concordant and discordant UWS and NP/OP groups with the Kruskal-Wallis test followed by a Dunn post-hoc test [16]. Also, the Chi-square test or Fisher’s exact test for selected categorical variables was conducted to assess differences in discordant results. All statistical analyses were performed using the software R, version 4.1.0 (R Core Team, 2021). Statistical significance was set at a *p*-value ≤ 0.05.

## Results

From January 3, 2022 to February 2, 2022, 365 outpatients were enrolled. The median age was 32.8 years, ranging from 3 to 94 years, with 111 (30.4%) patients under 18 years old. There were 221 (60.5%) female patients. A total of 119 (32.6%) patients had symptomatic, and 246 (67.4%) had an asymptomatic infection during the first sample collection. Selected baseline characteristics at enrollment are summarized in Table 1. None of the patients were hospitalized during the study period, and those who developed symptoms had mild or moderate disease. The median time between the onset of symptoms and the first sample collection was three days. Two participants developed symptoms in the interval between the two sample collections. RT-PCR for SARS-CoV-2 was confirmed in 97 out of 121 symptomatic patients (80.2%) either by detectable SARS-CoV-2 in NP/OP swab or UWS samples; most were adults (*n* = 71, 73.2%). Detectable RT-PCR for SARS-CoV-2 was observed in 62 out of 244 (25.4%) asymptomatic patients, and the majority also corresponded to adult patients (*n* = 44, 71.0%).

**Table 1:** Baseline characteristics of the participants enrolled in the study

Characteristics	Children and adolescents (< 18 years old) *n* = 111	Adults (18 years and older) *n* = 254
**Sex**		
Male	53 (47.7)	91 (35.8)
Female	58 (52.3)	163 (64.2)
**Age**		
3–12 years	74/365 (20.3)	-
≥12–18 years	37/365 (10.1)	-
≥18 years	-	254/365 (69.3)
Mean (SD)	10.2 (3.7)	43.7 (16.2)
Median (IQR)	9.7 (7.2, 13.4)	40.8 (31.7, 56.8)
[Minimum, Maximum]	[3.4, 17.5]	[18.3, 94.1]
**Highest educational attainment**		
Primary or lower	103 (92.8)	42 (16.5)
Secondary	8 (7.8)	102 (40.2)
University or postgraduate	-	101 (39.8)
Missing	-	9 (3.5)
**Main Comorbidities**		
Allergic rhinitis, yes	16 (14.4)	39 (15.4)
Asthma, yes	14 (12.6)	11 (4.3)
Hypertension, yes	-	66 (26.0)
Obesity, yes	-	41 (16.1)
Diabetes Mellitus, yes	-	18 (7.1)
Thyroid dysfunction, yes	-	18 (7.1)
Cardiovascular disease, yes	-	12 (4.7)
Cancer, yes	-	5 (2.0)
Kidney chronic disease, yes	-	3 (1.2)
HIV Infection, yes	-	2 (0.8)
Symptomatic infection, yes	33 (27.7)	86 (72.3)

*IQR* interquartile range, *SD * standard deviation

Overall, 818 samples were collected. Analysis of concordance was conducted between UWS and combined NP/OP samples showing an overall Cohen’s kappa value of 0.74 (95% CI: 0.67–0.81), indicating substantial agreement between the two sampling methods. Sensitivity was 77% (95% CI: 70.9–82.2), specificity 95% (95% CI: 91.9–97), PPV 89.8% (95% CI: 83.1–94.4), NPV 87.9% (95% CI: 83.6–91.5), and accuracy 88.5% (95% CI 85.0- 91.4) (Table 2).

**Table 2:** Overall results from combined nasopharyngeal/oropharyngeal swabs and unstimulated whole saliva samples in same-day matched pairs (*n* = 409)

	Combined nasopharyngeal/oropharyngeal swabs		
	Positive	Negative	Total
Unstimulated Whole Saliva	N (%)	N (%)	N (%)
**Positive**	114 (89.8)	13 (10.2)	127 (100)
**Negative**	34 (12.1)	248 (87.9)	282 (100)
**Total**	148 (36.2)	261 (63.8)	409 (100)

The comparison of performance between UWS and combined NP/OP samples, based on the RT-PCR detection of SARS-CoV-2 stratified by age groups and symptoms, is shown in Table 3.

**Table 3:** Summary of results obtained from parallel testing of combined nasopharyngeal/oropharyngeal swabs and unstimulated whole saliva stratified by age groups and symptoms (all samples)

	Combined nasopharyngeal/oropharyngeal swabs					
	Asymptomatic			Symptomatic		
**Unstimulated**	**Positive**	**Negative**	**Total**	**Positive**	**Negative**	**Total**
**Whole Saliva**	**N (%)**	**N (%)**	**N (%)**	**N (%)**	**N (%)**	**N (%)**
	**Children and adolescents (< 18 years old)**					
**Positive**	10 (83.3)	2 (16.7)	12 (100)	21 (95.5)	1 (4.5)	22 (100)
**Negative**	6 (8.1)	68 (91.9)	74 (100)	4 (36.4)	7 (63.6)	11 (100)
	**Adults (18 years and older)**					
**Positive**	30 (81.1)	7 (18.9)	37 (100)	53 (94.6)	3 (5.4)	56 (100)
**Negative**	7 (4.3)	156 (95.7)	163 (100)	17 (50.0)	17 (50.0)	34 (100)

Numbers in brackets stand for the proportion of the total of Unstimulated Whole Saliva for asymptomatic and symptomatic patients in each age group.

Sensitivity was higher among samples collected from symptomatic children and adolescent participants, and specificity was higher among asymptomatic patients regardless of age. The agreement between UWS and paired NP/OP swabs samples was substantial for asymptomatic and symptomatic children and adolescents, asymptomatic adults, and moderate for symptomatic adults (Table 4).

**Table 4:** Rates of sensitivity, specificity, positive predictive value, negative predictive value, accuracy, and Cohen’s kappa values of unstimulated whole saliva compared to combined nasopharyngeal/oropharyngeal swabs stratified by age group and symptoms (all samples)

	Asymptomatic	Symptomatic
	**Children and adolescents (< 18 years old)**	
**Sensitivity Estimate % (95% CI)**	62.5 (42.6–78.9)	84.0 (70.5–92.0)
**Specificity Estimate % (95% CI)**	97.1 (90.5–99.2)	87.5 (49.5–98.0)
**Positive Predictive Value Estimate % (95% CI)**	83.3 (51.6–97.9)	95.5 (77.2–99.9)
**Negative Predictive Value Estimate % (95% CI)**	91.9 (83.2–97.0)	63.6 (30.8–89.1)
**Accuracy Estimate % (95% CI)**	90.7 (82.5–95.9)	84.9 (68.1–94.9)
**Cohen’s kappa**^**a**^ **Estimate % (95% CI)**	0.66 (0.44–0.88)	0.63 (0.35–0.91)
	**Adults (18 years and older)**	
**Sensitivity Estimate % (95% CI)**	81.1 (67.1–90.0)	75.7 (68.9–81.4)
**Specificity Estimate % (95% CI)**	95.7 (92.0-97.8)	85.0 (64.2–94.7)
**Positive Predictive Value Estimate % (95% CI)**	81.1 (64.8–92.0)	94.6 (85.1–98.9)
**Negative Predictive Value Estimate % (95% CI)**	95.7 (91.4–98.3)	50.0 (32.4–67.6)
**Accuracy Estimate % (95% CI)**	93.0 (88.5–96.1)	77.8 (67.8–85.9)
**Cohen’s kappa**^**a**^ **Estimate % (95% CI)**	0.77 (0.65–0.88)	0.49 (0.30–0.67)

*CI* Confidence Interval, ^a^ Cohen’s kappa values: <0: No agreement; 0-0.20: Slight agreement; 0.21–0.40: Fair agreement; 0.41–0.60: Moderate agreement; 0.61–0.80: Substantial agreement; 0.81-1.0: Almost perfect agreement

**Fig. 1:**
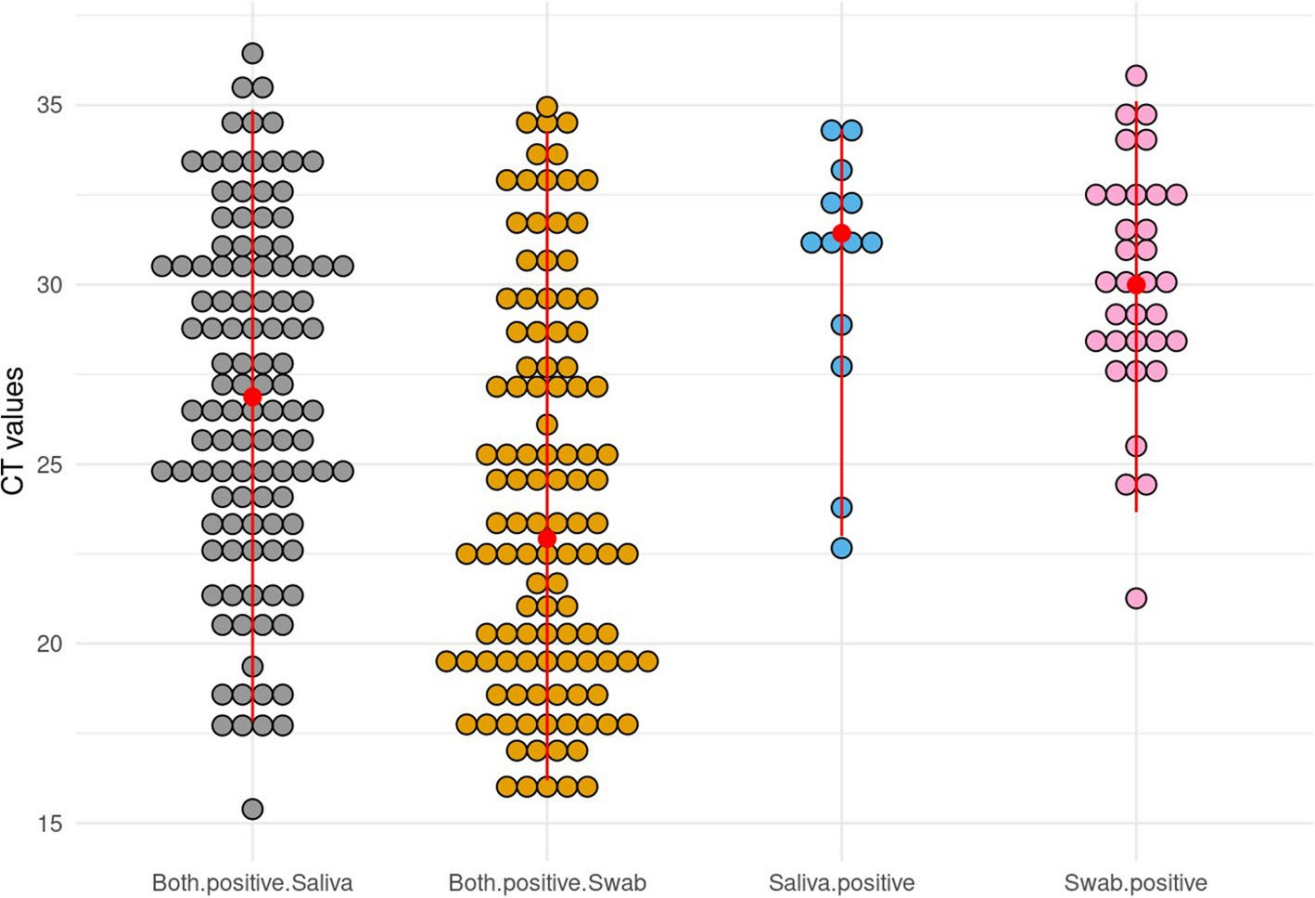
Cycle threshold values for E gene for unstimulated whole saliva and combined nasopharyngeal/oropharyngeal swabs. Distribution of Cycle threshold (Ct) values by testing concordance. Each “dot” represents a positive SARS-CoV-2 RT-PCR result. Red dots represent the median Ct values for both UWS and combined NP/OP swabs and positive for either UWS or combined NP/OP swabs. Data only include results of positive RT-PCR for SARS-CoV-2.

Overall, the distribution of Ct values of UWS (median, 27.7; IQR, 23.8–31.1) and paired NP/OP swabs (median, 25.2; IQR, 20.1–29.9) were different (*p* < 0.05). In addition, the distribution of Ct values from concordant results for UWS (median 26.9; IQR, 23.5–30.5) and paired NP/OP swabs (median 22.9; IQR, 19.5–27.6) was different (*p* < 0.05) (Fig. 1). However, there was no difference when the paired samples were discordant and positive only by UWS (median 31.4; IQR, 28.9–32.4) or by NP/OP swabs (median 30.0; IQR, 28.4–32.2), *p* = 0.99 (Fig. 1).

**Fig. 2:**
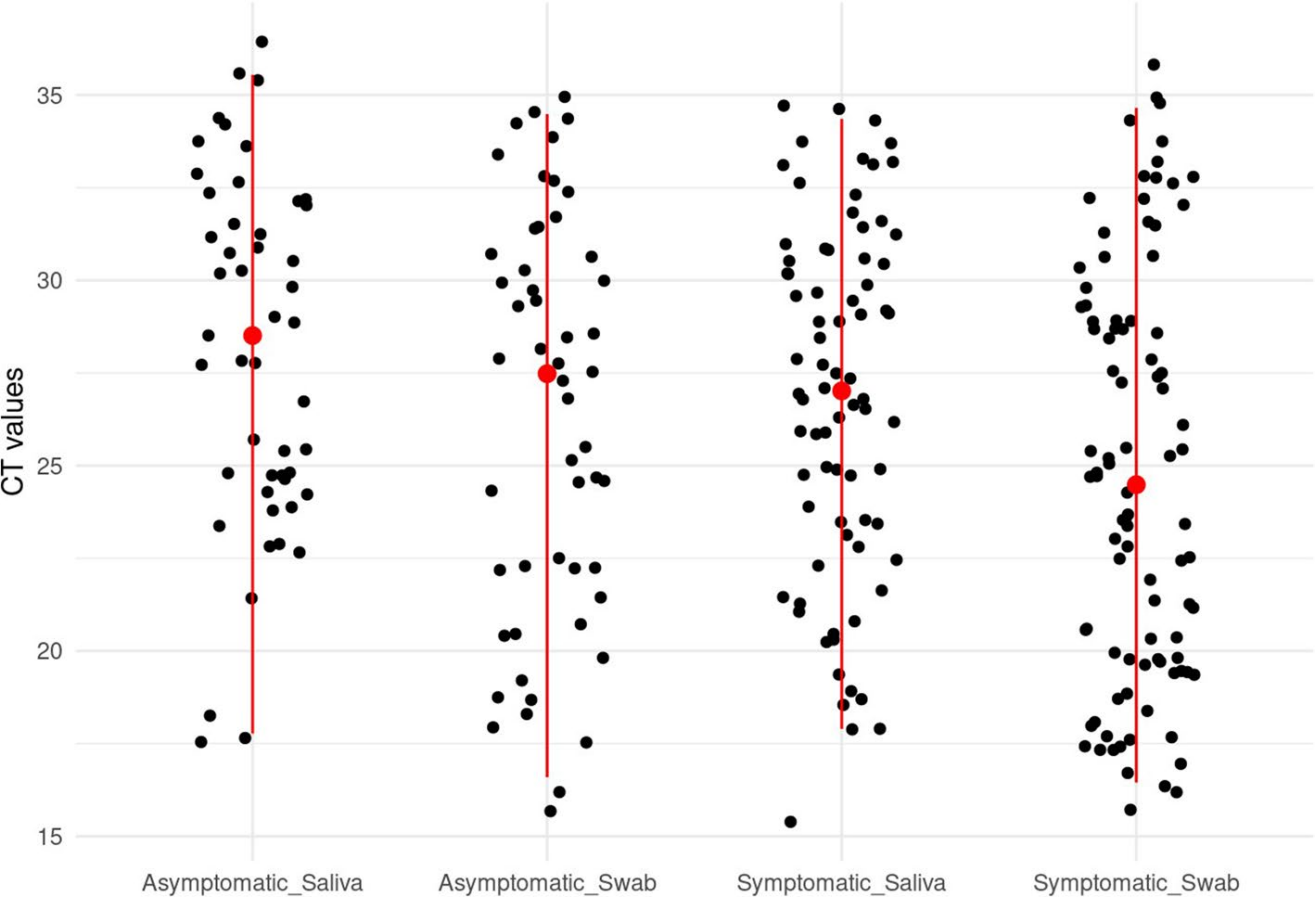
Comparison of Cycle threshold values of SARS-CoV-2 RT-PCR obtained from unstimulated whole saliva and combined nasopharyngeal/oropharyngeal swabs specimens for asymptomatic and symptomatic patients. Note. Cycle threshold (Ct) values are distributed by testing concordance between asymptomatic and symptomatic patients. Each “dot” represents a positive SARS-CoV-2 RT-PCR result. Red dots represent the median Ct values for unstimulated whole saliva and combined nasopharyngeal/oropharyngeal swabs. Data only include results of positive RT-PCR for SARS-CoV-2.

Moreover, no differences were observed between the UWS from asymptomatic patients (median 28.5; IQR, 24.6–32.0) and paired NP/OP (median 27.5; IQR, 22.2–30.6), *p* = 0.70. Marginal statistical significance was observed among paired samples from symptomatic patients (median 27.0; IQR, 23.4–30.5 for UWS and median 24.5; IQR, 19.8–28.9 for NP/OP), *p* = 0.08 (Fig. 2).

Selected sociodemographic and clinical characteristics of the participants with discordant results is shown in Table 5. No differences were observed between the groups.

**Table 5:** Sociodemographic and clinical characteristics of the participants with discordant results

Characteristics	Concordant results	Discordant results	*p*-value
	N (%)	N (%)	
**Sex**			
Male	128 (40.3)	16 (34.0)	0.416
Female	190 (59.7)	31 (66.0)	
**Age**			
<18 years	98 (30.8)	13 (27.7)	0.660
≥18 years	220 (69.2)	34 (72.3)	
**Highest educational attainment**			
Primary or lower	123 (39.7)	22 (47.8)	0.294
Secondary or higher	187(60.3)	24 (52.2)	
**Main Comorbidities**			
Allergic rhinitis (< 18 years)			
No	83 (84.7)	12 (92.3)	0.688*
Yes	15 (15.3)	1 (7.7)	
Asthma (< 18 years)			
No	85 (86.7)	12 (92.3)	1.000*
Yes	13 (13.3)	1 (7.7)	
Allergic rhinitis (≥ 18 years)			
No	185 (84.1)	30 (88.2)	0.533
Yes	35 (15.9)	4 (11.8)	
Asthma (≥ 18 years)			
No	210 (95.5)	33 (97.1)	1.000*
Yes	10 (4.5)	1 (2.9)	
Hypertension (≥ 18 years)			
No	164 (74.5)	24 (70.6)	0.624
Yes	56 (25.5)	10 (29.4)	
Obesity (≥ 18 years)			
No	188 (85.5)	25 (73.5)	0.079
Yes	32 (14.5)	9 (26.5)	
Diabetes Mellitus (≥ 18 years)			
No	203 (92.3)	33 (97.1)	0.482*
Yes	17 (7.7)	1 (2.9)	
Thyroid dysfunction (≥ 18 years)			
No	203 (92.3)	33 (97.1)	0.482*
Yes	17 (7.7)	1 (2.9)	

Highest educational attainment (missing = 9); *Fisher’s exact test

## Discussion

In this study, saliva samples had high sensitivity (84%) for the detection of SARS-CoV-2, with substantial agreement (Cohen’s kappa = 0.63) with combined NP/OP in symptomatic children and adolescents. In addition, saliva detected SARS-CoV-2 in 13 patients with negative combined NP/OP results.

Published studies comparing saliva with nasopharyngeal or oropharyngeal secretions to diagnose SARS-CoV-2 infection have shown conflicting results [7, 8, 17–26]. Banerjee et al. demonstrated high sensitivity (93%) and specificity (96.2%) of saliva specimens compared to paired NP swabs for SARS-CoV-2 detection in children (age range 5–18 years) [27]. Al Suwaidi et al., in a prospective study including 476 children (age range 3–18 years), suggested saliva as an alternative to upper respiratory tract samples for the diagnosis of SARS-CoV-2 [28].

We found that sensitivity was inferior for saliva in asymptomatic [62.5% (95% CI: 42.6–78.9)] compared with symptomatic [(84.0% (95% CI: 70.5–92.0)] children and adolescents. In contrast, Yee et al. reported that for symptomatic and asymptomatic pediatric patients not previously diagnosed with COVID-19, the performances of saliva and NP swabs were comparable (positive percent agreement: 82.4% versus 85.3%) [9].

Our results suggest that, according to the sensitivity estimates, saliva-based RT-PCR should be used with caution for asymptomatic SARS-CoV-2 infection screening during the circulation of the Omicron variant.

In asymptomatic and symptomatic adults, the sensitivity of saliva RT-PCR was 81.1% (95% CI: 67.1–90.0) and 75.7% (95% CI: 68.9–81.4), respectively. Similar results were reported by Pasomsub et al., who studied 200 sample pairs of nasopharyngeal and throat swabs and saliva samples from 200 individuals suspected to have COVID-19 [20]. In a prospective study with symptomatic outpatients, Landry et al. showed an overall sensitivity for SARS-CoV-2 detection of 85.7% for pure saliva compared to simultaneously-collected NP swabs [29]. Also, the median Ct value was significantly lower for NP swabs than for saliva.

A prospective study in symptomatic outpatients in Australia showed that 84.6% of patients with positive NP swabs had SARS-CoV-2 RNA detected in saliva, and 2% of saliva samples from patients with negative NP swabs were also positive [8]. Furthermore, the median Ct value was significantly lower in NP swabs than in saliva, suggesting a higher viral load in NP swabs [8]. Plantamura et al. also described Ct values as significantly lower in NP swabs than in saliva [30]. Similar Ct results were also observed in our study. On the other hand, Tutuncu et al. showed that mean Ct values of NP and saliva samples in mildly symptomatic and asymptomatic patients were not significantly different [31]. We may not have found a difference between the NP/OP and UWS samples of symptomatic participants due to the sample size of this group since marginal statistical significance was observed between paired NP/OP and UWS samples.

In our study, the overall Ct values in saliva were higher than those observed in the combined NP/OP swabs reflecting lower levels of viral nucleic acid, which may have impacted saliva sensitivity in the detection of SARS-CoV-2. The following factors may partially explain this difference: 1) When collecting saliva, patients accumulate saliva for one minute and then drool it into a sample container. In this way, some patients may not have provided a sufficient sample; 2) Combined collection of nasopharyngeal and oropharyngeal swabs may increase the concentration of viral particles in the sample, thereby increasing the sensitivity of NP/OP to UWS.

No sociodemographic and clinical characteristics evaluated were associated with the discordant combined NP/OP and UWS RT-PCR results.

The greatest strength of our study was a large cohort focusing on symptomatic and asymptomatic outpatients, including adults, children, and adolescents, and the opportunity to identify asymptomatic participants with the Omicron variant during regular cohort study visits and symptomatic participants during unscheduled visits.

Among the limitations of the study were: 1) Inclusion of only outpatients and not hospitalized participants; 2) Children under three were not enrolled; therefore, data cannot be generalized to this age group because saliva was collected by spontaneous production, not by swab collection; 3) Participants were enrolled in the household of an index case; therefore the pretest probability was high; 4) In the present study, there was good agreement between the Ct values of samples obtained via the two different sampling modes. On the other hand, the presence or absence of symptoms did not have a significant effect on Ct values. We may have failed to detect a difference in Ct values between symptomatic and asymptomatic patients due to insufficient statistical power.

## Conclusions

In summary, our findings suggest saliva is an adequate fluid for detecting SARS-CoV-2, especially for confirmation in symptomatic children and adolescents but should be used with caution for asymptomatic SARS-CoV-2 infection screening during the circulation of the Omicron variant. The negative predictive value was not high enough to exclude COVID-19, so a negative result should not be considered definitive, and the collection of additional samples is recommended especially in situations of high prevalence of COVID-19.

## Data Availability

Data underlying the study cannot be made publicly available due to ethical concerns, as data contain several personally identifiable information. Data are available from Oswaldo Cruz Foundation for researchers who meet the criteria for access to confidential data. Contact information: Institutional Ethics and Research Committee of the Evandro Chagas National Institute of Infectious Diseases, email: cep@ini.fiocruz.br or Guilherme Amaral Calvet; email: guilherme.calvet@ini.fiocruz.br.

## Abbreviations

CI: Confidence interval
COVID-19: Coronavirus disease, 2019
Ct: Cycle threshold
Fiocruz: Oswaldo Cruz Foundation
NP: Nasopharyngeal
NPV: Negative predictive value
OP: Oropharyngeal
PPV: Positive predictive value
RT-PCR: Real-time reverse transcriptase-polymerase chain reaction
SARS-CoV-2: Severe Acute Respiratory Syndrome Coronavirus 2
UWS: Unstimulated whole saliva
VTM: Viral Transport Medium
WHO: World Health Organization
VOC: Variant of concern
